# Feasibility and Efficacy of a Virtual Reality Game-Based Upper Extremity Motor Function Rehabilitation Therapy in Patients with Chronic Stroke: A Pilot Study

**DOI:** 10.3390/ijerph19063381

**Published:** 2022-03-13

**Authors:** Ángela Aguilera-Rubio, Alicia Cuesta-Gómez, Ana Mallo-López, Alberto Jardón-Huete, Edwin Daniel Oña-Simbaña, Isabel Mª Alguacil-Diego

**Affiliations:** 1 International PhD School, Rey Juan Carlos University, 28008 Madrid, Spain; a.aguilera.2016@alumnos.urjc.es (Á.A.-R.); a.mallo.2019@alumnos.urjc.es (A.M.-L.); 2 NeuroAvanza Neurological Physiotherapy Center, 28022 Madrid, Spain; 3 Department of Physical Therapy, Occupational Therapy, Rehabilitation and Physical Medicine, Faculty of Health Sciences, Rey Juan Carlos University, 28922 Madrid, Spain; isabel.alguacil@urjc.es; 4 Robotics Lab, University Carlos III of Madrid, Leganés, 28911 Madrid, Spain; ajardon@ing.uc3m.es (A.J.-H.); eona@ing.uc3m.es (E.D.O.-S.)

**Keywords:** stroke, virtual reality, upper extremity, video games, neurorehabilitation

## Abstract

Background: The objective of the present study was to develop a virtual reality protocol based on activities of daily living and conventional rehabilitation, using Leap Motion Controller to improve motor function in upper extremity rehabilitation in stroke patients. At the same time, the purpose was to explore its efficacy in the recovery of upper extremity motor function in chronic stroke survivors, and to determine feasibility, satisfaction and attendance rate; Methods: A prospective pilot experimental clinical trial was conducted. The outcome measures used were the grip strength, the Action Research Arm Test (ARAT), the Block and Box Test (BBT), the Short Form Health Survey-36 Questionnaire, a satisfaction questionnaire and attendance rate; Results: Our results showed statistically significant changes in the variables grip strength, BBT and ARAT as well as high levels of satisfaction and attendance; Conclusions: This virtual reality platform represents an effective tool in aspects of upper extremity functionality rehabilitation in patients with chronic stroke, demonstrating feasibility and high levels of attendance and satisfaction.

## 1. Introduction

Stroke is the leading cause of acquired disability in adults. It is a pathology that implies a health, personal, family and social burden due to its impact on the lives of the people who suffer from it and their caregivers [1].

The involvement of the upper extremity (UE) appears in up to 85% of patients who survive a stroke, affecting their quality of life [2]. The recovery of the motor function of the UE is essential in the development of ADLs, but it is also the origin of the difficulty of their recovery, as many of these activities require the coordinated use of both hands [3]. Furthermore, the complexity of its recovery, compared to the lower limb, underlies the fact that the main functional objective of the UE is the interaction between the individual and the environment in an efficient way, and for this, it is necessary to be able to move the hand in space performing reaching movements, subsequently being able to manipulate, grasp and develop various activities [3,4].

Rehabilitation treatment after a stroke often requires a long period of time, in some cases even a lifetime, and motivation plays a key role in this process. This motivation is the most important factor in terms of treatment effect in stroke patients, so treatment effects depend not only on the efforts of doctors and therapists, but also on the patient’s active participation, willingness and motivation. Hence, the lower the patient’s motivation in this process, the more difficult the rehabilitation treatment becomes [5,6].

The continuous challenge of functional recovery from MS has led to different techniques and approaches being developed in the field of neurorehabilitation. In recent years, virtual reality (VR) has become part of rehabilitation protocols as an additional tool, enabling the development of necessary goals in the rehabilitation process such as task orientation, repetition and the possibility of creating intensive treatments [7,8]. VR offers patients solutions on both physical and psychological levels, both common problems in this pathology [9]. Some authors predict that VR glasses therapies will become a broad field of research encompassing medical, technological and psychological disciplines in the coming years [9].

In stroke patients (in acute, sub-acute and chronic phases), the benefits of VR are demonstrated when it is used as a complement to another intervention, and its results are not inferior to other therapies when used as the single treatment. It offers benefits such as: the opportunity to interact in virtual environments similar to reality, through multi-sensory integration (visual, auditory, tactile), greater patient motivation and participation, as well as being increasingly economical, thus popularizing its use [2,6]. Despite their increasing use, there is a lack of games specifically designed for stroke patients. Commercial games do not take into consideration the peculiarities of these patients such as increased tone, among others [8], and therefore, specific protocols for this population are necessary.

Leap Motion Controller (LMC) is a low-cost VR device, recently used in the field of neurorehabilitation [10,11], that does not use motion markers, and collects forearm, wrist and hand displacements. LMC uses two cameras and three infrared light-emitting diodes (LEDs, wavelength 850 nm) to track palm position, wrist orientation and the five digits. Thanks to its wide-angle lens, the device features a large interaction space of eight cubic feet, which takes the shape of an inverted pyramid [12]. The collected data are transmitted via USB to the LMC tracking software. This system analyzes the images to reconstruct a 3D representation of what the device sees. The result is the generation of a semi-immersive virtual environment, where the subject is introduced into this environment thanks to a pre-presentation of his or her body or part of it, called the virtual identity or avatar [13].

The objective of the proposed pilot study was to develop a VR protocol based on activities of daily living and conventional rehabilitation, using LMC-based serious games to improve motor function in UE rehabilitation in stroke patients; to explore the preliminary efficacy of LMC in the recovery of motor function of the UE in survivors of a chronic stroke; and to determine the feasibility, satisfaction and attendance rate of the use of this therapeutic VR platform in a rehabilitation program for the recovery of the UE after stroke.

## 2. Materials and Methods

### 2.1. Pilot Study

#### 2.1.1. Participants

All subjects had to meet the following inclusion criteria: adults with stroke of more than 6 months of evolution, able to maintain a sitting position independently without back support and with a score equal to or greater than 16 on the Fugl Meyer scale of UE function.

The patients who presented in any of these exclusion criteria were not selected for the study: people with an added diagnosis of other pathologies that limit occupational performance, with cognitive impairment that affects the ability to understand language to follow instructions, with cerebellar disorders, with hemineglect and hemianopsia and/or visual disturbances that cannot be corrected with ocular devices.

#### 2.1.2. Procedure

This study protocol was approved by a local ethics committee. The ethical principles for medical research in humans of the Declaration of Helsinki were followed. Informed consent was obtained from each subject after they had been provided with a detailed explanation of the objectives of the study and the procedures to be used.

The participants received 2 sessions of 60 min duration per week, for eight weeks, distributed as follows: 30 min of conventional physiotherapy treatment (based on shoulder, elbow, wrist and finger mobilization, strengthening of UE extensor muscles, stretching exercises for UE flexor muscles, exercises to improve motor control of UE) plus 30 min of VR. Two evaluations were carried out: pre-treatment and post-treatment, immediately after finishing the eighth week of treatment. The intervention took place in the Association of Stroke Sufferers (Rehabictus) in Leganés (Madrid, Spain).

### 2.2. Design and Protocol

The LMC device was used to capture the movement of the UE of stroke patients, and a virtual environment consisting of four games specifically designed by engineers from the Robotics Laboratory of the Carlos III University using the Unity3D Game Engine software.

The serious games performed were intended to imitate exercises and movements commonly included in conventional rehabilitation. The games were specifically designed for these patients, and focused on improving UE functionality through movements that this population usually has altered (proximal stability of the shoulder girdle, all movements involving the shoulder joint, elbow flexion-extension, forearm pronation-supination, wrist and finger flexion-extension and palmar grasp).

All games were first played unilaterally (first the sound hand in order to familiarize with the game, then the affected hand), and finally with both hands simultaneously, except for the game known as “sequence”.

Due to the heterogeneity of UE symptomatology in stroke patients, the games were easy to be customized according to the patients’ needs and skill level. For each of the games, the number of fruits that appear initially in the screen, distances between them and how far and close they will be placed could be tuned, and those settings were stored in a configuration file associated with the patient’s ID. The settings could be defined by therapists at the beginning of the training session, or during the performance of the video game, and were recorded into a file with the time score and failure in case of timeout. To avoid user frustration, the therapist could press whenever a skip button (the green right button) to set the task as completed.

**Figure 1:**
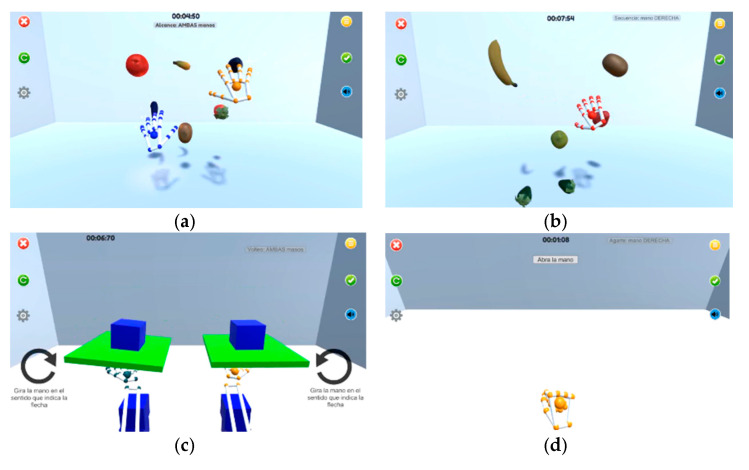
(**a**) Reach game; (**b**) Sequence game; (**c**) Flip game; (**d**) Opening/closing game.

The characteristics of the four games were (an image of the games is shown in Figure 1):

Reach game: In this game, several fruit-shaped objects were shown within the reaching range of the user’s UE represented in different locations. The user had to touch the fruit that is highlighted. Once the fruit was reached, the gravity was activated so it falls to the floor of the virtual scene. To complete the game, the user had to reach all cubes shown in random order (Figure 1a)

Sequence game: This game used the same set-up as the Reach Game. Now a sequence of fruits was presented to the user, who had to memorize the sequence and repeat it by reaching the fruits in the same order shown, adding a cognitive training to the game (Figure 1b).

Flip game: This game trained pronation and supination movements of the forearm. The user had to place the palm of the hand over the LMC device imitating a waiter holding out a tray. A small tray with a cube in the middle appeared in the center of the screen. The patient then had to turn the palm downwards. Upon doing so, the cube to detach from the tray and fall to the ground (Figure 1c).

Opening/closing game: this game encouraged the user to achieve the opening and/or closing of the hand, simulating grasping movements, depending on the percentage specifically programmed for each of them. A red circle in the centre of the screen was shown to indicate to the user where to place the fruits into. When a fruit was highlighted, the user had to grasp it and move it to the red circle while keeping their hand closed, and keep this gesture until the hand touched the red circle. Then the user had to open the hand (Figure 1d).

The participant received both visual and auditory instructions to carry out the objective of each game. In addition, the therapist remained close by, in case any clarification or help was needed, especially in the first sessions.

**Figure 2:**
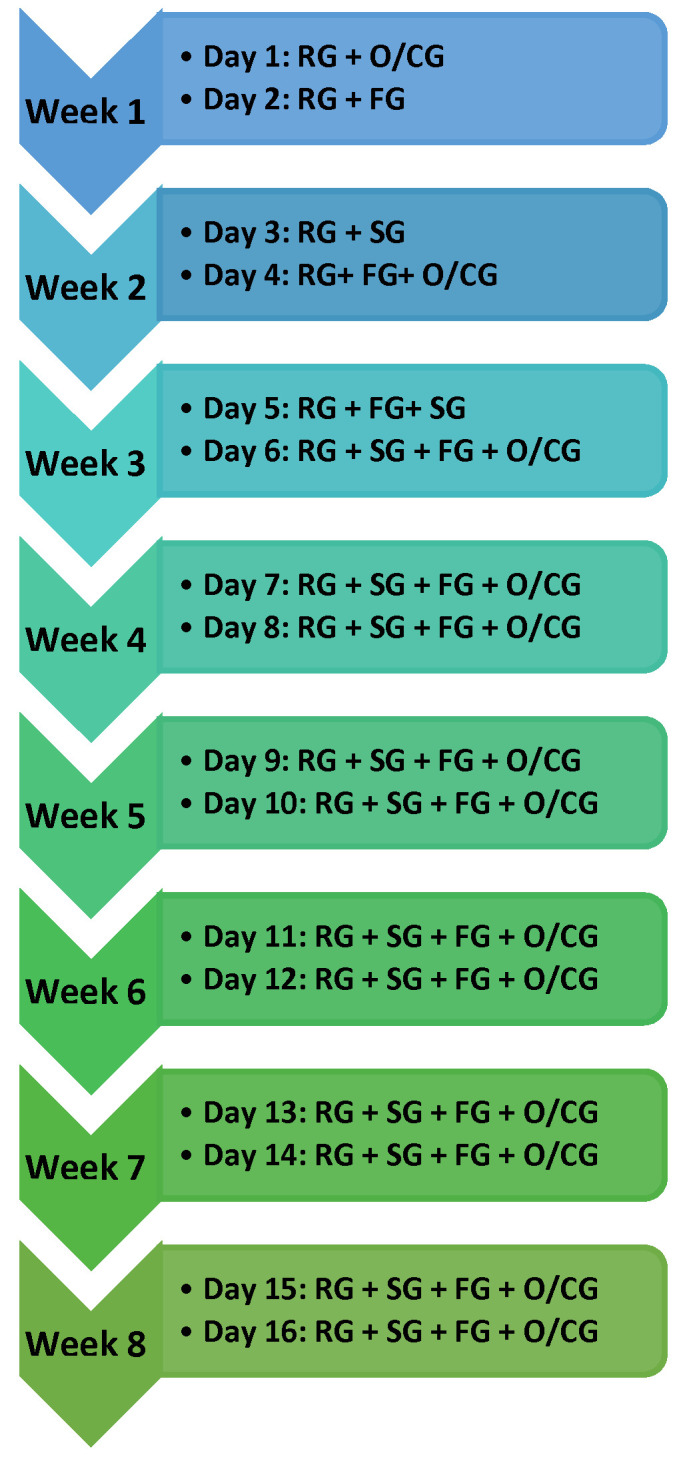
Experimental protocol. RG: reach game; SG: sequence game; FP: flip game; O/CG: opening/closing game.

The protocol used in this study is shown in Figure 2.

Following a stroke, alterations in muscle tone, strength, coordination, sensation and pain can limit the movement of the UE in the reaching pattern. Because of this reduced mobility, many patients rely on the trunk to perform compensatory movements in flexion and rotation to complete the reaching task [14,15]. Cirstea et al. [16] observed a significant correlation between decreased elbow extension and shoulder flexion and increased trunk movement in stroke patients.

**Figure 3:**
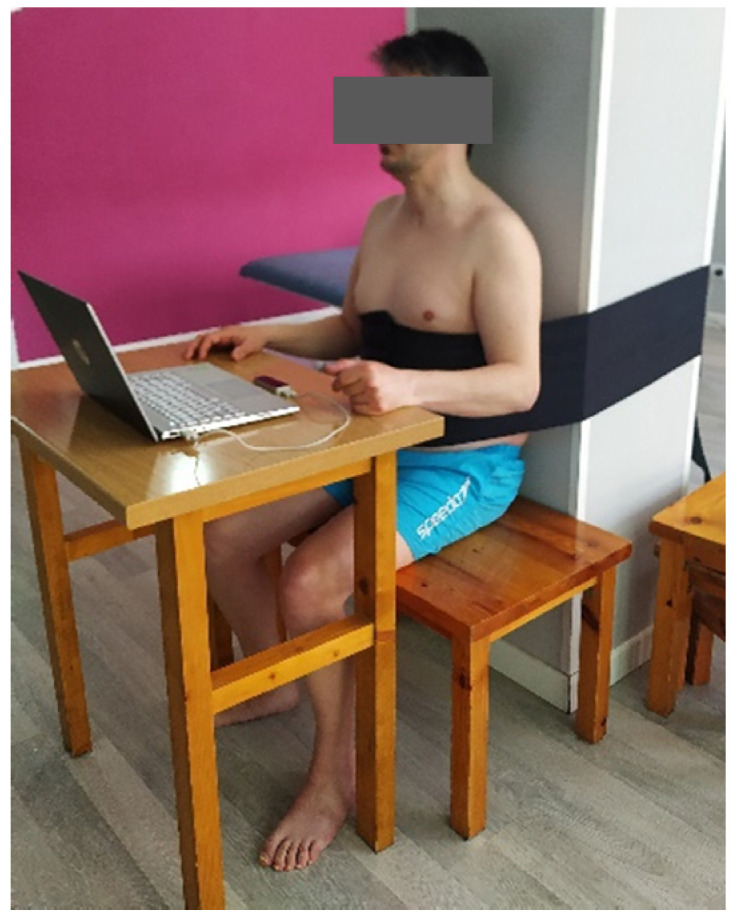
Position of the patient.

In order to try to control this compensation, and given that all patients had trunk control, the protocol includes performing the intervention in a seated position, and trunk restraint with an elastic strap (Figure 3) in an attempt to facilitate the development of typical motor patterns in the affected UE, as was proposed by other authors in the treatment of stroke patients [14,17,18,19,20,21,22].

#### 2.2.1. Outcome Measures

Before the beginning of the treatment protocol and after its completion, the following outcome measures were evaluated:

- Grip strength. A hydraulic Jamar hand dynamometer was used to measure the maximum grip strength. This was conducted according to the protocol of the American Hand Therapy Association [23]. In a seated position, participants had the arm in adduction, the elbow flexed to 90 degrees, the forearm in neutral position and the wrist between 0 and 30 degrees of extension. Three consecutive trials were carried out with the affected hand, and the mean of the three trials was used for statistical analysis. Clinically, grip strength is an important factor, as in the elderly population, it can inform future development as well as the most appropriate therapeutic approach [24]. Bertrand et al. [24] measured their test-retest reality, concluding that it was excellent (ICC 0.80 to 0.89).
- Action Research Arm Test (ARAT). It is a reliable tool for assessing UE motor deficits after stroke. It is a recommended test for stroke patients with an excellent test-retest reliability for this population (ICC = 0.965) [25]. It consists of 19 tests subdivided into 4 groups (grasp, grip, pinch and gross movement). The higher the score, the better the UE motor skills [26].
- The Box and Blocks Test (BBT). Through this test, gross manual dexterity was measured, both on the more affective and the less affective side. The BBT consists of moving, for one minute and one at a time, the maximum number of blocks from one side of a box to the other. It is an objective, easy to perform, reliable and standardized test and is validated for both acute and chronic stroke patients [27,28]. The minimum detectable change in stroke patients is 1.99 blocks in the most affected hand, and 2.84 blocks in the less affected hand. It has an excellent test-retest reliability for both the most affected hand (r = 0.98) and the least affected hand (r = 0.93) [28].
- Short Form Health Survey-36 Questionnaire (SF-36). The SF-36 is a generic scale on health status, valid both in the general population and in certain groups such as stroke. It consists of 36 questions subdivided into 8 scales: Physical Function, Physical Role, Body Pain, General Health, Vitality, Social Function, Emotional Role and Mental Health. It also adds an item comparing one’s health status with that of the previous year. It is used in descriptive studies as well as in therapeutic interventions [29,30]. In patients with chronic stroke, it has an excellent interrater/intra-rater reality with an ICC = 0.89 [31].
- Patient satisfaction was assessed by means of a questionnaire based on a Likert-type scale, designed by the research group. The questionnaire is made up of 9 items that assess the usefulness of the LMC in their rehabilitation, the degree of motivation, possible technical problems during the intervention, usability, whether they reported pain, the importance of therapist support, experience, frequency of use of electronic devices and the use of new technologies in the rehabilitation process. The range of the questionnaire was from 1 to 4, and the maximum possible score was 36. All questions were directly proportional, i.e., the higher the score, the better the patient’s perception [32,33].
- Attendance rate. Additionally, we recorded the attendance rate (%) for therapy sessions (compliance).

#### 2.2.2. Data Analysis

Statistical analysis was performed using the SPSS statistical software system (version 22.0, SPSS Inc., Chicago, IL, USA). Being a small sample, it was verified whether the variables followed a normal distribution through the Shapiro–wilk test. The hypothesis that the variables did not have a normal distribution was accepted, due to the result of the test and the verification of the histograms of each variable. The Wilcoxon test, a non-parametric test for related samples, was used. The statistical analysis was carried out with a confidence level of 95%, for which reason that those whose *p* was < 0.05 were considered significant values.

## 3. Results

A total of 10 participants were recruited, 5 male and 5 female. All were ischemic strokes except one, which was hemorrhagic. The age of the patients ranged from 32 to 75 years, with a mean age of 59.50 (±11.43) years. In two of the patients, the left cerebral hemisphere was affected, while for the rest it was the right cerebral hemisphere. The evolution time was 10 ± 7 years.

The statistical analysis pre-intervention and post-intervention showed statistically significant changes in grip strength, ARAT total and BBT. Significant improvements were observed on the grip strength (*p* = 0.005), the ARAT total (*p* = 0.028) and the BBT (*p* = 0.011). These results mean that patients improved their scores in post-treatment measurements (Table 1).

Related to the satisfaction questionnaire, the patients presented a high degree of satisfaction with the LMC device and the proposed rehabilitation protocol, obtaining a mean score of 30.00 (±3.01) (Table 2).

No side effects were observed, except for two patients who reported shoulder pain, compatible with tendon overload, which did not prevent them from continuing with the exercise program. Finally, 100% attendance rate to treatment was observed.

## 4. Discussion

The aim of the present study was to develop a VR protocol with games designed ad hoc with the LMC device to improve the functionality of the UE in patients with chronic stroke. In addition, objectives were to present preliminary results of its efficacy and to determine feasibility, satisfaction and attendance rate of use.

In recent years, there were a few studies on the use of the LMC system in VR environments to improve the functionality of the UE in stroke patients [34,35,36,37,38,39]. Most of these studies were applied in acute [35,38] and sub-acute phases [34,37,38,39], and only one study applied it to chronic stroke patients [36]. Moreover, in all of them, it is not indicated whether the games used were commercially or purposely designed. The variable spontaneous recovery that occurs in the first months after stroke may be a confounding factor in rehabilitation intervention [40]. During this period, it is difficult to quantify the patient’s improvement in relation to the therapeutic intervention. On the other hand, after the first 3 months, recovery seems to depend mainly on the different strategies for adapting to learning [40].

Some patients respond to specific treatments later in life, well beyond the subacute phase, and rehabilitation treatment should be maintained as long as there are functional goals to be achieved [41]. Teasell et al [42] also suggest, following a compilation of chronic phase research, that the rehabilitation of many stroke patients reflects a lifelong learning process.

Stroke is an age-dependent pathology, but current records also show a number of cases in an increasingly younger population [1]. Although our sample reflects the current situation in society in terms of the age of onset of stroke, it is an aspect that makes it difficult to homogenize the samples.

The rehabilitation process in this phase is carried out on an ambulatory stage, and it is necessary that patients continue to have access to rehabilitation services. The goal in the chronic phase is to promote independence and reintegration of patients into society, but there is little provision of therapy-based rehabilitation services for patients more than one year after stroke [43].

Knowing that improvements in the UE in stroke patients are not only limited to the first months, but appear even after many years of evolution [40,42], together with the lack of literature found on the use of LMC in chronic patients [44], and the difficulty of accessing rehabilitation services in these phases, justify the need for studies of these characteristics.

Regarding the number of sessions, Laver et al. [7] in their review suggested that VR interventions with more than 15 h in total were the best option, as well as tailored interventions. Other authors, such as Aramaki et al. [45], emphasize the predominance of short and moderate duration and low intensity protocols in terms of weekly sessions. The intervention methodology of most of the studies analyzed in their review was to apply two sessions per week, lasting between 30 and 60 min each, over six weeks. Likewise, it seems that the amount of therapy with the LMC device is not a determining factor in the improvement of the functionality of the UE, as significant improvements were obtained with different times [34,35,36].

The heterogeneity of methods and instruments used in studies of VR in stroke patients sometimes hinders the evaluation of the efficacy of this therapy. In the review of Bui et al [3], it is concluded that the combination of VR with conventional therapy may be particularly effective in the treatment of the UE in stroke. Another review [46], on the other hand, justifies that the use of VR does not lead to improvements in manual dexterity in stroke patients.

Compared to studies that have used LMC, our preliminary results on the functional aspects of the UE are in line with those of some authors. The BBT showed significant improvements in line with Fluet et al. [39]; grip strength also reached significance, similar to other studies [34,35]; and finally, ARAT scores achieved statistical significance as in the research of Ögün et al [36]. No quality of life questionnaire was used in previous studies, and although in our research we analyzed the general quality of life questionnaire SF-36, perhaps, as no significant changes were observed, it may be necessary in future trials to add specific quality of life questionnaires analyzing quality of life in relation to the UE. Even so, it is necessary to take into account the lack of consensus in all these studies, in terms of time and dose of treatment, complementary use or not to other therapy, as well as common criteria for quantifying the degree of dysfunctionality of the UE to serve as a starting point [44].

Lack of motivation is often common in long-term rehabilitation processes such as stroke [5]. The high attendance rates observed not only in our research, but also in those that implement VR [36,47], seem to promote its use for the improvement of functional tasks in stroke rehabilitation.

Regarding the satisfaction questionnaire, the question with the lowest score was the one referring to technical problems during the use of the LMC, such as loss of signal among others. This problem was also reported by other authors [34], attributing it to the fact that the optical sensors of the device show certain problems in following the movement of the fingers when the hands are overlapped, or when the participant has a high level of spasticity. These technical problems cause the patient to feel frustrated, which may even decrease motivation. On the other hand, the questions with the highest scores were the intention to incorporate new technologies in the rehabilitation process and the question about pain. It seems necessary to take this last aspect into account, as it is estimated that the incidence of shoulder pain in stroke patients is as high as 84% [48], and it is significant that other authors have not reflected this [34,35,36,37,38,39].

Although it is not the aim of this research, it seems interesting to include additional lines of research in future studies. On the one hand, to analyze by means of functional MRI whether this protocol produces brain changes in terms of cognitive aspects such as attention or memory, since the games involve these concepts, beyond motor rehabilitation, especially in the sequence game. Other authors already used imaging to test functional changes after the use of LMC in subacute patients [37]. In addition, the characteristics of this device, its low cost, small size and ease of use [13], make it a possible device to be used in home treatment, even in telerehabilitation protocols. Vanbellingen et al. [35] already reflected the interest of patients to continue treatment at home, and Fluet et al. [39] developed their VR rehabilitation system at home. Finally, it should be noted that in this case, the feedback is only visual, and perhaps a glove could be introduced to add sensory feedback, as other authors have implemented [49].

Some 44% of the total cost of stroke in Europe is direct health care spending (hospital care and medication); estimates of new cases of stroke in Europe are 34%, and the prevalence rate is increasing steadily. This represents a growing challenge for a society that needs to meet the long-term needs of stroke survivors [50]. Lee et al. [51] in their systematic review report that the implementation of such virtual reality devices in neurorehabilitation is safe and cost-effective, resulting in a reduction in long-term healthcare costs. Therefore, the high burden of stroke treatment justifies trying to implement such devices in new treatment approaches so that chronic patients, after discharge, can continue therapy in a fun and motivating way.

Although our results may be encouraging, this study has several limitations that need to be mentioned. Firstly, they cannot be extended to all stroke patients, since neither the time of evolution nor the degree of UE involvement is the same, and therefore, they must be interpreted with caution. Another important limitation is the small sample size, the sample heterogeneity (not all patients had the same type of stroke, which may cause a bias in the results presented), the lack of follow-up and the absence of a control group to assess the effectiveness of this device. An additional limitation that should be noted is that an evaluation of the cognitive deterioration of the patients was not included, and the satisfaction questionnaire was created by the researchers, and its reliability has not been evaluated. Therefore, randomized clinical trials with larger sample sizes and follow-up evaluation are needed to verify these preliminary results. Finally, technical adjustments to the device are needed to ensure proper image reception.

## 5. Conclusions

The LMC system and the VR protocol presented in this study represent an effective tool in aspects of UE functionality in patients with chronic stroke. It showed improvements in grip strength, manual dexterity and functional recovery of the UE. This VR platform demonstrated feasibility and high levels of attendance and motivation. However, future research is needed to verify these results.

## Figures and Tables

**Table 1:** Comparison Outcome measures.

Variable	Pre	Post	*p* Value
Grip strength	16.30 (13.20)	17.80 (14.90)	0.005 *
ARAT total	48 (30)	50 (31)	0.028 *
ARAT A	14 (8)	16 (9)	0.139
ARAT B	12 (5)	12 (5)	0.157
ARAT C	12 (15)	13 (13)	0.168
ARAT D	9 (2)	9 (1)	0.157
BBT	27 (24)	20.50 (23)	0.011 *
SF-36. Physical function	45 (37.50)	50 (40)	0.655
SF36. Physical role	62.50 (100)	62.50 (100)	0.317
SF-36. Body ache	35 (30)	30 (27.50)	0.059
SF-36. General health	60 (13.80)	60 (13.80)	0.317
SF-36. Vitality	55 (13.80)	57.50 (13.80)	0.317
SF-36. Social function	50 (12.50)	50 (24.90)	0.083
SF-36. Emotional role	100 (91.80)	100 (66.90)	0.317
SF-36. Mental health	62 (11)	62 (12)	0.197

ARAT: Action Research Arm Test; BBT: Box and Block Test; SF-36: Short Form Health Survey-36 Questionnaire. Data are expressed as median and interquartile range. * *p* Value < 0.05 using the Wilcoxon test for related samples.

**Table 2:** Patients’ satisfaction.

Item	Media (Standard Deviation)
Motivation	3.50 (0.527)
Usability	3.00 (0.816)
Use of electronic devices	2.70 (0.949)
Pain	3.80 (0.422)
Therapist support	3.70 (0.483)
Technical problems	2.60 (0.966)
Utility	3.50 (0.527)
Experience	3.40 (0.516)
New technologies	3.80 (0.422)

## Data Availability

Not applicable.
